# *catena*-Poly[[[diaqua­cobalt(II)]bis­(μ-1,3-di-4-pyridylpropane-κ^2^ *N*:*N*′)] bis­(perchlorate) bis­(1,3-di-4-pyridyl­propane) bis­(2-methyl-4-nitro­aniline)]

**DOI:** 10.1107/S1600536808026470

**Published:** 2008-08-23

**Authors:** Zhiyong Fu, Yuan Chen, Jianglong Yi

**Affiliations:** a School of Chemistry and Chemical Engineering, South China University of Technology, Guangzhou, People’s Republic of China

## Abstract

In the title compound, {[Co(C_13_H_14_N_2_)_2_(H_2_O)_2_](ClO_4_)_2_·2C_13_H_14_N_2_·2C_7_H_8_N_2_O_2_}_*n*_, the Co^II^ ion lies on a crystallographic inversion center and is coordinated by four N atoms from four symmetry-related 1,3-di-4-pyridylpropane ligands and two O atoms from two water ligands in a slightly distorted octa­hedral coordination environment. The 1,3-di-4-pyridylpropane ligands are doubly bridging and connect the Co^II^ ions into one-dimensional chains. The asymmetric unit also contains one uncoordinated 1,3-di-4-pyridylpropane mol­ecule, one 2-methyl-4-nitro­aniline mol­ecule and one perchlorate anion. In the crystal structure, inter­molecular O—H⋯N hydrogen bonds connect the one-dimensional chains into a two-dimensional network.

## Related literature

For a related complex with a similar crystal structure, see: Merz *et al.* (2004 ▶). For related literature, see: James (2003 ▶). 
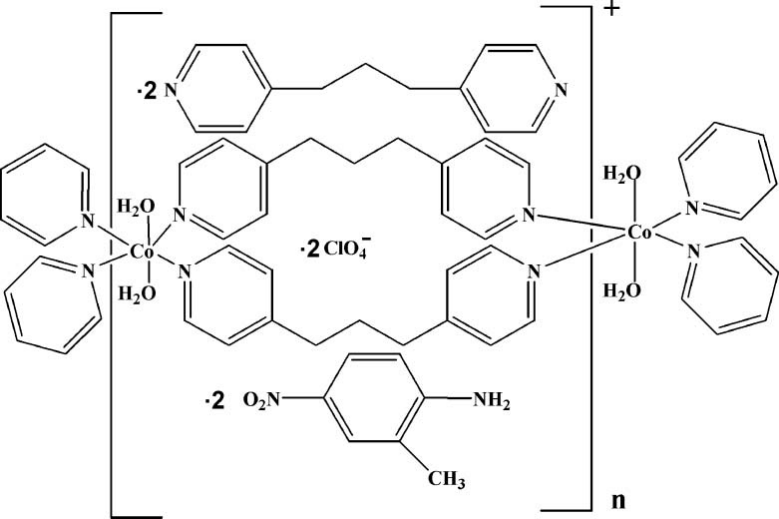


## Experimental

### 

#### Crystal data

[Co(C_13_H_14_N_2_)_2_(H_2_O)_2_](ClO_4_)_2_·2C_13_H_14_N_2_·2C_7_H_8_N_2_O_2_
*M* *_r_* = 1391.22
Triclinic, 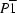

*a* = 10.9310 (3) Å
*b* = 11.6505 (5) Å
*c* = 15.2054 (4) Å
α = 71.0112 (7)°
β = 82.4011 (6)°
γ = 68.3933 (7)°
*V* = 1702.22 (10) Å^3^
*Z* = 1
Mo *K*α radiation
μ = 0.40 mm^−1^
*T* = 298 (2) K
0.30 × 0.22 × 0.1 mm

#### Data collection

Bruker SMART CCD diffractometer
Absorption correction: multi-scan (*SADABS*; Sheldrick, 1996 ▶) *T*_min_ = 0.892, *T*_max_ = 0.967
8612 measured reflections
5827 independent reflections
3438 reflections with *I* > 2σ(*I*)
*R*_int_ = 0.039

#### Refinement

*R*[*F* ^2^ > 2σ(*F* ^2^)] = 0.066
*wR*(*F* ^2^) = 0.168
*S* = 1.02
5827 reflections
438 parameters
H atoms treated by a mixture of independent and constrained refinement
Δρ_max_ = 0.53 e Å^−3^
Δρ_min_ = −0.31 e Å^−3^

### 

Data collection: *SMART* (Bruker, 1996 ▶); cell refinement: *SAINT* (Bruker, 1996 ▶); data reduction: *SAINT*; program(s) used to solve structure: *SHELXS97* (Sheldrick, 2008 ▶); program(s) used to refine structure: *SHELXL97* (Sheldrick, 2008 ▶); molecular graphics: *SHELXTL* (Sheldrick, 2008 ▶); software used to prepare material for publication: *SHELXTL*.

## Supplementary Material

Crystal structure: contains datablocks global, I. DOI: 10.1107/S1600536808026470/lh2679sup1.cif

Structure factors: contains datablocks I. DOI: 10.1107/S1600536808026470/lh2679Isup2.hkl

Additional supplementary materials:  crystallographic information; 3D view; checkCIF report

## Figures and Tables

**Table:** 

Co1—O1	2.090 (3)
Co1—N2^i^	2.179 (3)
Co1—N1	2.208 (3)

**Table:** 

O1^ii^—Co1—O1	180
O1^ii^—Co1—N2^i^	92.94 (13)
O1—Co1—N2^i^	87.06 (13)
N2^iii^—Co1—N2^i^	180
O1^ii^—Co1—N1	88.90 (12)
O1—Co1—N1	91.10 (12)
N2^iii^—Co1—N1	87.20 (11)
N2^iii^—Co1—N1^ii^	92.80 (11)
N1—Co1—N1^ii^	180

Symmetry codes: (i) 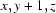; (ii) 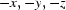; (iii) 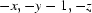.

**Table 2:** Hydrogen-bond geometry (Å, °)

*D*—H⋯*A*	*D*—H	H⋯*A*	*D*⋯*A*	*D*—H⋯*A*
O1—H1⋯N3^iii^	0.85 (6)	1.94 (3)	2.744 (5)	173 (4)
O1—H2⋯N4^iv^	0.73 (4)	2.07 (2)	2.810 (3)	174 (3)

Symmetry codes: (iii) 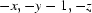; (iv) 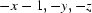.

**Fig. 1.**
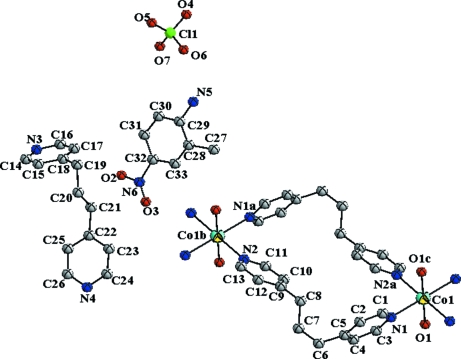
The asymmetric unit of (I) with some symmetry related atoms. Displacement ellipsoids are drawn at the 50% probability level [Symmetry Codes: (a) -x, -1-y, -z, (b) x, -1+y, z, (c) -x, -y, -z].

**Fig. 2.**
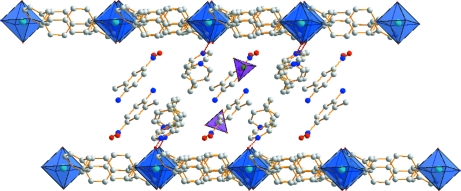
The packing of (I), viewed approximately along the a axis, showing the O—H···N hydrogen bonds between the guest 4,4,-trimethylenedipyridine molecule and the coordinated water molecules.

## supplementary crystallographic information

### Comment

Crystal engineering of metal organic frameworks has advanced dramatically over the past decade as a result of the number of interesting structural motifs (James, 2003). As part of our investigation of the use of 4,4,-trimethylenedipyridine as ligand in the construction of new coordination polymers, the title compound was obtained as a salt. The crystal structure of the title compound (I) comprises [Co(4,4,-trimethylenedipyridine)_2_(H_2_O)_2_]_n_ polymeric chains, solvate 2-methyl-4-nitroaniline molecules, solvate 4,4,-trimethylenedipyridine molecules, and perchlorate anions. The asymmetric unit of (I) plus some symmetry related atoms are shown in Fig. 1. Each Co^II^ ion is in a slightly distorted octahedral environment. The equatorial plane consists of four nitrogen atoms from 4,4,-trimethylenedipyridine (Co—N 2.177 (4)–2.208 (4) Å) and the axial positions are occupied by two aqua molecules (Co—O 2.109 (3) Å). The Co^II^ ions, separated by approximately 11.65 Å, are bridged by 4,4,-trimethylenedipyridine ligands forming a polymeric chain. The structure possesses a distorted macrocycle enclosed by 4,4,-trimethylenedipyridine liaginds. The dimensions of the distorted square cavity are approximately 11.65*4.36 Å, measured from opposite phenyl ring to phenyl ring. The distorted macrocyclic structure includes low-symmetry guest molecules. The solvated 2-methyl-4-nitroaniline molecules solvated in the structure interact with the rest of the structure by normal van der Waals forces. There are two types of 4,4,-trimethylenedipyridine ligands located in the crystal structure. One is coordinated to the Co center while the other is a guest molecule. In the crystal structure, O—H···N hydrogen bonds exist between the water molecules and the guest 4,4,-trimethylenedipyridine molecules. Figure 2 shows the packing in (I) the extended two-dimensional network.

### Experimental

An aqueous mixture (10 ml) containing 4,4,-trimethylenedipyridine (0.1 g, 0.5 mmol), Co(ClO_4_)_2_(H_2_O)_6_ (0.365 g, 1 mmol), 2-methyl-4-nitroaniline (0.075 g, 0.5 mmol) was placed in a Parr Teflonlined stainless steel vessel(25 ml), and the vessel was sealed and heated to 388.15 K for 24 h. 0.090 g Orange sheet-like crystals were obtained.

### Refinement

H atoms bonded to the O atoms of the water molecules were located in a difference map and refined with distance restraints of O—H = 0.85 (3) Å. Other H atoms were positioned geometrically and refined using a riding model, with C—H = 0.93–0.97 Å and with *U*_iso_(H) = 1.2 (1.5 for methyl groups) times *U*_eq_(C).

### Crystal data

**Table:**

[Co(C_13_H_14_N_2_)_2_(H_2_O)_2_](ClO_4_)_2_·2C_13_H_14_N_2_·2C_7_H_8_N_2_O_2_	*Z* = 1
*M**_r_* = 1391.22	*F*_000_ = 729
Triclinic, *P*1	*D*_x_ = 1.357 Mg m^−^^3^
Hall symbol: -P 1	Mo *K*α radiation λ = 0.71073 Å
*a* = 10.9310 (3) Å	Cell parameters from 5827 reflections
*b* = 11.6505 (5) Å	θ = 2.8–25.0º
*c* = 15.2054 (4) Å	µ = 0.40 mm^−^^1^
α = 71.0112 (7)º	*T* = 298 (2) K
β = 82.4011 (6)º	Block, orange
γ = 68.3933 (7)º	0.30 × 0.22 × 0.1 mm
*V* = 1702.22 (10) Å^3^	

### Data collection

**Table:**

Bruker SMART CCD diffractometer	5827 independent reflections
Radiation source: fine-focus sealed tube	3438 reflections with *I* > 2σ(*I*)
Monochromator: graphite	*R*_int_ = 0.039
*T* = 298(2) K	θ_max_ = 25.0º
ω scans	θ_min_ = 2.8º
Absorption correction: Multi-scan(SADABS; Sheldrick, 1996)	*h* = −11→13
*T*_min_ = 0.892, *T*_max_ = 0.967	*k* = −13→13
8612 measured reflections	*l* = −18→18

### Refinement

**Table:**

Refinement on *F*^2^	Secondary atom site location: difference Fourier map
Least-squares matrix: full	Hydrogen site location: inferred from neighbouring sites
*R*[*F*^2^ > 2σ(*F*^2^)] = 0.066	H atoms treated by a mixture of independent and constrained refinement
*wR*(*F*^2^) = 0.168	*w* = 1/[σ^2^(*F*_o_^2^) + (0.0705*P*)^2^] where *P* = (*F*_o_^2^ + 2*F*_c_^2^)/3
*S* = 1.02	(Δ/σ)_max_ < 0.001
5827 reflections	Δρ_max_ = 0.53 e Å^−^^3^
438 parameters	Δρ_min_ = −0.31 e Å^−^^3^
Primary atom site location: structure-invariant direct methods	Extinction correction: none

### Special details

**Table:**

Refinement. Refinement of *F*^2^ against ALL reflections. The weighted *R*-factor *wR* and goodness of fit *S* are based on *F*^2^, conventional *R*-factors *R* are based on *F*, with *F* set to zero for negative *F*^2^. The threshold expression of *F*^2^ > σ(*F*^2^) is used only for calculating *R*-factors(gt) *etc*. and is not relevant to the choice of reflections for refinement. *R*-factors based on *F*^2^ are statistically about twice as large as those based on *F*, and *R*- factors based on ALL data will be even larger.

### Fractional atomic coordinates and isotropic or equivalent isotropic displacement parameters (Å^2^)

**Table:**

	*x*	*y*	*z*	*U*_iso_*/*U*_eq_	
Co1	0.0000	0.0000	0.0000	0.0368 (2)	
O1	−0.0308 (3)	−0.0246 (3)	0.1425 (2)	0.0450 (8)	
H2	−0.095 (4)	0.007 (4)	0.161 (3)	0.035 (14)*	
H1	−0.009 (5)	−0.091 (6)	0.190 (4)	0.10 (2)*	
O2	0.0102 (8)	−0.3950 (9)	−0.2105 (4)	0.185 (3)	
O3	−0.1176 (7)	−0.1911 (10)	−0.2256 (7)	0.218 (5)	
O4	0.6660 (4)	−0.3125 (5)	−0.7027 (3)	0.1267 (17)	
O5	0.5089 (6)	−0.3630 (6)	−0.7540 (3)	0.146 (2)	
O6	0.4479 (6)	−0.1872 (5)	−0.7051 (4)	0.160 (2)	
O7	0.5165 (4)	−0.3851 (5)	−0.5995 (3)	0.1177 (16)	
N1	−0.1793 (3)	−0.0409 (3)	−0.0082 (2)	0.0380 (8)	
N2	−0.1087 (3)	−0.7946 (3)	−0.0213 (2)	0.0412 (8)	
N3	−0.0498 (4)	−0.7448 (4)	−0.2812 (3)	0.0679 (11)	
N4	−0.7219 (4)	−0.0999 (4)	−0.2125 (3)	0.0621 (10)	
N5	0.3025 (6)	−0.1123 (7)	−0.5321 (4)	0.139 (2)	
H5A	0.3638	−0.1666	−0.5550	0.167*	
H5B	0.2891	−0.0309	−0.5548	0.167*	
N6	−0.0249 (11)	−0.2734 (10)	−0.2435 (5)	0.156 (4)	
C1	−0.2391 (4)	−0.0147 (3)	−0.0875 (3)	0.0422 (10)	
H1A	−0.2139	0.0379	−0.1416	0.051*	
C2	−0.3361 (4)	−0.0616 (4)	−0.0933 (3)	0.0464 (10)	
H2A	−0.3749	−0.0399	−0.1501	0.056*	
C3	−0.2207 (4)	−0.1150 (4)	0.0678 (3)	0.0499 (11)	
H3A	−0.1830	−0.1329	0.1243	0.060*	
C4	−0.3148 (4)	−0.1659 (4)	0.0676 (3)	0.0507 (11)	
H4A	−0.3386	−0.2177	0.1229	0.061*	
C5	−0.3749 (4)	−0.1408 (4)	−0.0143 (3)	0.0455 (11)	
C6	−0.4719 (4)	−0.2046 (4)	−0.0158 (3)	0.0528 (11)	
H6A	−0.5153	−0.1653	−0.0754	0.063*	
H6B	−0.5386	−0.1897	0.0321	0.063*	
C7	−0.4055 (4)	−0.3506 (4)	0.0001 (3)	0.0521 (11)	
H7A	−0.3683	−0.3907	0.0619	0.062*	
H7B	−0.4714	−0.3867	−0.0025	0.062*	
C8	−0.2983 (4)	−0.3827 (4)	−0.0706 (3)	0.0528 (11)	
H8A	−0.3356	−0.3428	−0.1323	0.063*	
H8B	−0.2325	−0.3462	−0.0680	0.063*	
C9	−0.2331 (4)	−0.5255 (4)	−0.0547 (3)	0.0426 (10)	
C10	−0.1315 (4)	−0.5980 (4)	0.0076 (3)	0.0513 (11)	
H10A	−0.1023	−0.5576	0.0398	0.062*	
C11	−0.0732 (4)	−0.7292 (4)	0.0225 (3)	0.0488 (11)	
H11A	−0.0055	−0.7749	0.0652	0.059*	
C12	−0.2711 (4)	−0.5940 (4)	−0.0986 (3)	0.0484 (10)	
H12A	−0.3400	−0.5508	−0.1405	0.058*	
C13	−0.2081 (4)	−0.7249 (4)	−0.0807 (3)	0.0450 (10)	
H13A	−0.2360	−0.7676	−0.1117	0.054*	
C14	−0.1616 (6)	−0.7095 (5)	−0.3234 (4)	0.0786 (15)	
H14A	−0.2033	−0.7700	−0.3105	0.094*	
C15	−0.2205 (5)	−0.5909 (5)	−0.3846 (4)	0.0716 (14)	
H15A	−0.2992	−0.5733	−0.4120	0.086*	
C16	0.0060 (5)	−0.6559 (5)	−0.3035 (4)	0.0723 (14)	
H16A	0.0856	−0.6768	−0.2760	0.087*	
C17	−0.0468 (5)	−0.5330 (5)	−0.3655 (4)	0.0681 (14)	
H17A	−0.0019	−0.4751	−0.3792	0.082*	
C18	−0.1640 (4)	−0.4972 (4)	−0.4060 (3)	0.0536 (11)	
C19	−0.2286 (5)	−0.3641 (5)	−0.4704 (3)	0.0738 (14)	
H19A	−0.2426	−0.3711	−0.5298	0.089*	
H19B	−0.1699	−0.3154	−0.4809	0.089*	
C20	−0.3608 (6)	−0.2899 (5)	−0.4324 (4)	0.0908 (18)	
H20A	−0.3999	−0.2071	−0.4786	0.109*	
H20B	−0.4196	−0.3383	−0.4226	0.109*	
C21	−0.3491 (5)	−0.2670 (5)	−0.3435 (4)	0.0829 (16)	
H21A	−0.2998	−0.2092	−0.3551	0.099*	
H21B	−0.3000	−0.3485	−0.2997	0.099*	
C22	−0.4832 (5)	−0.2082 (5)	−0.2996 (3)	0.0655 (13)	
C23	−0.5308 (5)	−0.0799 (5)	−0.3042 (3)	0.0755 (15)	
H23A	−0.4834	−0.0265	−0.3359	0.091*	
C24	−0.6487 (5)	−0.0307 (5)	−0.2618 (3)	0.0696 (14)	
H24A	−0.6797	0.0573	−0.2677	0.084*	
C25	−0.5612 (5)	−0.2796 (5)	−0.2533 (4)	0.0686 (14)	
H25A	−0.5359	−0.3659	−0.2511	0.082*	
C26	−0.6763 (5)	−0.2240 (5)	−0.2102 (3)	0.0642 (13)	
H26A	−0.7251	−0.2757	−0.1776	0.077*	
C27	0.1226 (7)	0.0718 (7)	−0.4533 (5)	0.120 (2)	
H27A	0.0538	0.1196	−0.4192	0.180*	
H27B	0.1029	0.1068	−0.5184	0.180*	
H27C	0.2046	0.0781	−0.4435	0.180*	
C28	0.1332 (6)	−0.0690 (6)	−0.4197 (4)	0.0822 (16)	
C29	0.2250 (6)	−0.1562 (7)	−0.4586 (4)	0.0839 (17)	
C30	0.2475 (6)	−0.2857 (7)	−0.4235 (5)	0.0873 (17)	
H30A	0.3157	−0.3425	−0.4485	0.105*	
C31	0.1723 (7)	−0.3344 (6)	−0.3523 (5)	0.0910 (18)	
H31A	0.1872	−0.4226	−0.3277	0.109*	
C32	0.0689 (6)	−0.2398 (8)	−0.3182 (4)	0.086 (2)	
C33	0.0569 (6)	−0.1127 (7)	−0.3516 (4)	0.0845 (17)	
H33A	−0.0073	−0.0540	−0.3257	0.101*	
Cl1	0.53712 (14)	−0.31378 (14)	−0.69036 (9)	0.0756 (4)	

### Atomic displacement parameters (Å^2^)

**Table:**

	*U*^11^	*U*^22^	*U*^33^	*U*^12^	*U*^13^	*U*^23^
Co1	0.0321 (4)	0.0319 (4)	0.0486 (5)	−0.0089 (3)	0.0030 (4)	−0.0188 (4)
O1	0.0393 (19)	0.0465 (19)	0.0476 (19)	−0.0105 (15)	0.0078 (16)	−0.0203 (17)
O2	0.257 (8)	0.263 (9)	0.105 (4)	−0.181 (8)	0.027 (5)	−0.054 (5)
O3	0.150 (6)	0.336 (12)	0.284 (9)	−0.104 (6)	0.096 (6)	−0.257 (9)
O4	0.084 (3)	0.198 (5)	0.098 (3)	−0.081 (3)	0.011 (3)	−0.014 (3)
O5	0.168 (5)	0.224 (6)	0.110 (4)	−0.110 (5)	0.030 (3)	−0.097 (4)
O6	0.159 (5)	0.099 (4)	0.183 (5)	−0.025 (3)	0.006 (4)	−0.017 (4)
O7	0.121 (3)	0.154 (4)	0.077 (3)	−0.079 (3)	0.013 (3)	−0.001 (3)
N1	0.0388 (19)	0.0339 (18)	0.0442 (19)	−0.0108 (14)	0.0062 (16)	−0.0202 (15)
N2	0.0374 (19)	0.0380 (19)	0.0479 (19)	−0.0095 (15)	0.0041 (16)	−0.0185 (16)
N3	0.057 (3)	0.063 (3)	0.067 (3)	−0.008 (2)	0.009 (2)	−0.016 (2)
N4	0.041 (2)	0.073 (3)	0.068 (3)	−0.005 (2)	−0.001 (2)	−0.033 (2)
N5	0.126 (5)	0.208 (7)	0.109 (4)	−0.096 (5)	0.034 (4)	−0.051 (4)
N6	0.242 (10)	0.174 (8)	0.120 (6)	−0.154 (9)	−0.045 (7)	−0.020 (6)
C1	0.043 (2)	0.034 (2)	0.051 (3)	−0.0155 (18)	0.001 (2)	−0.0131 (19)
C2	0.043 (2)	0.034 (2)	0.065 (3)	−0.0071 (19)	−0.007 (2)	−0.023 (2)
C3	0.054 (3)	0.055 (3)	0.049 (3)	−0.026 (2)	0.002 (2)	−0.019 (2)
C4	0.049 (3)	0.059 (3)	0.057 (3)	−0.032 (2)	0.011 (2)	−0.024 (2)
C5	0.028 (2)	0.032 (2)	0.081 (3)	−0.0046 (17)	0.004 (2)	−0.030 (2)
C6	0.029 (2)	0.049 (3)	0.088 (3)	−0.0095 (19)	0.005 (2)	−0.037 (2)
C7	0.043 (2)	0.037 (2)	0.084 (3)	−0.0168 (19)	0.009 (2)	−0.029 (2)
C8	0.062 (3)	0.032 (2)	0.062 (3)	−0.013 (2)	0.011 (2)	−0.019 (2)
C9	0.039 (2)	0.033 (2)	0.053 (2)	−0.0119 (18)	0.012 (2)	−0.0160 (19)
C10	0.049 (3)	0.044 (3)	0.068 (3)	−0.014 (2)	0.003 (2)	−0.031 (2)
C11	0.046 (3)	0.041 (2)	0.060 (3)	−0.0072 (19)	−0.009 (2)	−0.023 (2)
C12	0.044 (2)	0.036 (2)	0.062 (3)	−0.0081 (19)	−0.004 (2)	−0.015 (2)
C13	0.043 (2)	0.041 (2)	0.058 (3)	−0.0158 (19)	0.004 (2)	−0.024 (2)
C14	0.074 (4)	0.072 (4)	0.095 (4)	−0.030 (3)	0.005 (3)	−0.028 (3)
C15	0.058 (3)	0.081 (4)	0.077 (4)	−0.015 (3)	−0.010 (3)	−0.030 (3)
C16	0.047 (3)	0.084 (4)	0.078 (4)	−0.018 (3)	−0.005 (3)	−0.017 (3)
C17	0.055 (3)	0.066 (3)	0.082 (4)	−0.025 (3)	0.004 (3)	−0.018 (3)
C18	0.044 (3)	0.058 (3)	0.050 (3)	−0.003 (2)	0.008 (2)	−0.025 (2)
C19	0.066 (3)	0.072 (3)	0.058 (3)	−0.002 (3)	0.011 (3)	−0.018 (3)
C20	0.090 (4)	0.081 (4)	0.061 (3)	0.015 (3)	−0.004 (3)	−0.019 (3)
C21	0.066 (4)	0.084 (4)	0.085 (4)	−0.003 (3)	−0.004 (3)	−0.033 (3)
C22	0.052 (3)	0.075 (4)	0.061 (3)	−0.006 (3)	0.005 (2)	−0.029 (3)
C23	0.074 (4)	0.073 (4)	0.069 (3)	−0.018 (3)	0.017 (3)	−0.024 (3)
C24	0.067 (3)	0.063 (3)	0.070 (3)	−0.005 (3)	0.009 (3)	−0.032 (3)
C25	0.057 (3)	0.060 (3)	0.083 (4)	−0.001 (3)	−0.007 (3)	−0.035 (3)
C26	0.051 (3)	0.075 (4)	0.065 (3)	−0.017 (3)	−0.005 (3)	−0.023 (3)
C27	0.131 (6)	0.113 (6)	0.127 (6)	−0.042 (5)	−0.030 (5)	−0.038 (5)
C28	0.073 (4)	0.100 (5)	0.078 (4)	−0.024 (4)	−0.012 (4)	−0.036 (4)
C29	0.073 (4)	0.120 (6)	0.078 (4)	−0.045 (4)	0.000 (3)	−0.041 (4)
C30	0.074 (4)	0.104 (5)	0.096 (5)	−0.027 (4)	0.004 (4)	−0.052 (4)
C31	0.099 (5)	0.094 (5)	0.101 (5)	−0.041 (4)	−0.019 (4)	−0.040 (4)
C32	0.089 (5)	0.156 (7)	0.051 (3)	−0.083 (5)	0.005 (3)	−0.034 (4)
C33	0.065 (4)	0.119 (5)	0.091 (4)	−0.032 (4)	0.003 (3)	−0.061 (4)
Cl1	0.0767 (10)	0.0836 (10)	0.0635 (8)	−0.0338 (8)	0.0127 (7)	−0.0164 (7)

### Geometric parameters (Å, °)

**Table:**

Co1—O1^i^	2.090 (3)	C9—C12	1.385 (5)
Co1—O1	2.090 (3)	C10—C11	1.374 (5)
Co1—N2^ii^	2.179 (3)	C10—H10A	0.9300
Co1—N2^iii^	2.179 (3)	C11—H11A	0.9300
Co1—N1	2.208 (3)	C12—C13	1.371 (5)
Co1—N1^i^	2.208 (3)	C12—H12A	0.9300
O1—H2	0.73 (4)	C13—H13A	0.9300
O1—H1	0.85 (6)	C14—C15	1.362 (7)
O2—N6	1.263 (10)	C14—H14A	0.9300
O3—N6	1.183 (12)	C15—C18	1.375 (6)
O4—Cl1	1.402 (4)	C15—H15A	0.9300
O5—Cl1	1.390 (4)	C16—C17	1.389 (6)
O6—Cl1	1.400 (5)	C16—H16A	0.9300
O7—Cl1	1.400 (4)	C17—C18	1.358 (6)
N1—C3	1.336 (5)	C17—H17A	0.9300
N1—C1	1.340 (4)	C18—C19	1.500 (6)
N2—C11	1.337 (5)	C19—C20	1.534 (7)
N2—C13	1.338 (5)	C19—H19A	0.9700
N2—Co1^iv^	2.179 (3)	C19—H19B	0.9700
N3—C14	1.319 (6)	C20—C21	1.490 (7)
N3—C16	1.320 (6)	C20—H20A	0.9700
N4—C24	1.333 (6)	C20—H20B	0.9700
N4—C26	1.334 (6)	C21—C22	1.537 (7)
N5—C29	1.387 (7)	C21—H21A	0.9700
N5—H5A	0.8600	C21—H21B	0.9700
N5—H5B	0.8600	C22—C23	1.371 (7)
N6—C32	1.481 (10)	C22—C25	1.377 (7)
C1—C2	1.383 (5)	C23—C24	1.372 (7)
C1—H1A	0.9300	C23—H23A	0.9300
C2—C5	1.382 (5)	C24—H24A	0.9300
C2—H2A	0.9300	C25—C26	1.375 (6)
C3—C4	1.365 (5)	C25—H25A	0.9300
C3—H3A	0.9300	C26—H26A	0.9300
C4—C5	1.381 (6)	C27—C28	1.516 (8)
C4—H4A	0.9300	C27—H27A	0.9600
C5—C6	1.508 (5)	C27—H27B	0.9600
C6—C7	1.531 (5)	C27—H27C	0.9600
C6—H6A	0.9700	C28—C33	1.329 (8)
C6—H6B	0.9700	C28—C29	1.368 (8)
C7—C8	1.508 (6)	C29—C30	1.363 (8)
C7—H7A	0.9700	C30—C31	1.370 (8)
C7—H7B	0.9700	C30—H30A	0.9300
C8—C9	1.498 (5)	C31—C32	1.436 (8)
C8—H8A	0.9700	C31—H31A	0.9300
C8—H8B	0.9700	C32—C33	1.361 (8)
C9—C10	1.383 (5)	C33—H33A	0.9300
			
O1^i^—Co1—O1	180	C12—C13—H13A	118.2
O1^i^—Co1—N2^ii^	87.06 (13)	N3—C14—C15	124.7 (5)
O1—Co1—N2^ii^	92.94 (13)	N3—C14—H14A	117.6
O1^i^—Co1—N2^iii^	92.94 (13)	C15—C14—H14A	117.6
O1—Co1—N2^iii^	87.06 (13)	C14—C15—C18	120.3 (5)
N2^ii^—Co1—N2^iii^	180	C14—C15—H15A	119.8
O1^i^—Co1—N1	88.90 (12)	C18—C15—H15A	119.8
O1—Co1—N1	91.10 (12)	N3—C16—C17	124.1 (5)
N2^ii^—Co1—N1	87.20 (11)	N3—C16—H16A	118.0
N2^iii^—Co1—N1	92.80 (11)	C17—C16—H16A	118.0
O1^i^—Co1—N1^i^	91.10 (12)	C18—C17—C16	120.0 (5)
O1—Co1—N1^i^	88.90 (12)	C18—C17—H17A	120.0
N2^ii^—Co1—N1^i^	92.80 (11)	C16—C17—H17A	120.0
N2^iii^—Co1—N1^i^	87.20 (11)	C17—C18—C15	115.8 (4)
N1—Co1—N1^i^	180	C17—C18—C19	122.4 (5)
Co1—O1—H2	121 (3)	C15—C18—C19	121.7 (4)
Co1—O1—H1	132 (4)	C18—C19—C20	112.9 (4)
H2—O1—H1	95 (5)	C18—C19—H19A	109.0
C3—N1—C1	115.8 (3)	C20—C19—H19A	109.0
C3—N1—Co1	118.7 (3)	C18—C19—H19B	109.0
C1—N1—Co1	124.6 (3)	C20—C19—H19B	109.0
C11—N2—C13	116.0 (3)	H19A—C19—H19B	107.8
C11—N2—Co1^iv^	120.4 (3)	C21—C20—C19	113.4 (4)
C13—N2—Co1^iv^	123.6 (2)	C21—C20—H20A	108.9
C14—N3—C16	115.0 (4)	C19—C20—H20A	108.9
C24—N4—C26	115.2 (4)	C21—C20—H20B	108.9
C29—N5—H5A	120.0	C19—C20—H20B	108.9
C29—N5—H5B	120.0	H20A—C20—H20B	107.7
H5A—N5—H5B	120.0	C20—C21—C22	113.0 (4)
O3—N6—O2	130.4 (11)	C20—C21—H21A	109.0
O3—N6—C32	120.4 (10)	C22—C21—H21A	109.0
O2—N6—C32	109.2 (10)	C20—C21—H21B	109.0
N1—C1—C2	123.7 (4)	C22—C21—H21B	109.0
N1—C1—H1A	118.2	H21A—C21—H21B	107.8
C2—C1—H1A	118.2	C23—C22—C25	116.3 (5)
C5—C2—C1	119.6 (4)	C23—C22—C21	120.8 (5)
C5—C2—H2A	120.2	C25—C22—C21	122.9 (5)
C1—C2—H2A	120.2	C24—C23—C22	119.7 (5)
N1—C3—C4	124.0 (4)	C24—C23—H23A	120.1
N1—C3—H3A	118.0	C22—C23—H23A	120.1
C4—C3—H3A	118.0	N4—C24—C23	124.7 (5)
C3—C4—C5	120.2 (4)	N4—C24—H24A	117.7
C3—C4—H4A	119.9	C23—C24—H24A	117.7
C5—C4—H4A	119.9	C26—C25—C22	120.4 (5)
C4—C5—C2	116.7 (3)	C26—C25—H25A	119.8
C4—C5—C6	120.3 (4)	C22—C25—H25A	119.8
C2—C5—C6	123.0 (4)	N4—C26—C25	123.6 (5)
C5—C6—C7	112.2 (3)	N4—C26—H26A	118.2
C5—C6—H6A	109.2	C25—C26—H26A	118.2
C7—C6—H6A	109.2	C28—C27—H27A	109.5
C5—C6—H6B	109.2	C28—C27—H27B	109.5
C7—C6—H6B	109.2	H27A—C27—H27B	109.5
H6A—C6—H6B	107.9	C28—C27—H27C	109.5
C8—C7—C6	112.7 (3)	H27A—C27—H27C	109.5
C8—C7—H7A	109.1	H27B—C27—H27C	109.5
C6—C7—H7A	109.1	C33—C28—C29	117.9 (6)
C8—C7—H7B	109.1	C33—C28—C27	122.1 (7)
C6—C7—H7B	109.1	C29—C28—C27	120.0 (7)
H7A—C7—H7B	107.8	C30—C29—C28	121.7 (6)
C9—C8—C7	112.7 (3)	C30—C29—N5	119.2 (7)
C9—C8—H8A	109.1	C28—C29—N5	119.0 (7)
C7—C8—H8A	109.1	C29—C30—C31	121.8 (6)
C9—C8—H8B	109.1	C29—C30—H30A	119.1
C7—C8—H8B	109.1	C31—C30—H30A	119.1
H8A—C8—H8B	107.8	C30—C31—C32	115.3 (6)
C10—C9—C12	115.6 (3)	C30—C31—H31A	122.3
C10—C9—C8	121.1 (4)	C32—C31—H31A	122.3
C12—C9—C8	123.3 (4)	C33—C32—C31	120.2 (5)
C11—C10—C9	120.7 (4)	C33—C32—N6	116.6 (8)
C11—C10—H10A	119.7	C31—C32—N6	123.2 (8)
C9—C10—H10A	119.7	C28—C33—C32	122.8 (6)
N2—C11—C10	123.5 (4)	C28—C33—H33A	118.6
N2—C11—H11A	118.3	C32—C33—H33A	118.6
C10—C11—H11A	118.3	O5—Cl1—O6	107.3 (4)
C13—C12—C9	120.6 (4)	O5—Cl1—O7	110.2 (3)
C13—C12—H12A	119.7	O6—Cl1—O7	107.4 (3)
C9—C12—H12A	119.7	O5—Cl1—O4	111.1 (3)
N2—C13—C12	123.6 (4)	O6—Cl1—O4	109.6 (3)
N2—C13—H13A	118.2	O7—Cl1—O4	111.0 (3)

Symmetry codes: (i) −*x*, −*y*, −*z*; (ii) −*x*, −*y*−1, −*z*; (iii) *x*, *y*+1, *z*; (iv) *x*, *y*−1, *z*.

### Hydrogen-bond geometry (Å, °)

**Table:**

*D*—H···*A*	*D*—H	H···*A*	*D*···*A*	*D*—H···*A*
O1—H1···N3^ii^	0.85 (6)	1.94 (3)	2.744 (5)	173 (4)
O1—H2···N4^v^	0.73 (4)	2.07 (2)	2.810 (3)	174 (3)

Symmetry codes: (ii) −*x*, −*y*−1, −*z*; (v) −*x*−1, −*y*, −*z*.
